# Recent advances in proximity-based labeling methods for interactome mapping

**DOI:** 10.12688/f1000research.16903.1

**Published:** 2019-01-31

**Authors:** Laura Trinkle-Mulcahy

**Affiliations:** 1 Department of Cellular and Molecular Medicine and Ottawa Institute of Systems Biology, Faculty of Medicine, University of Ottawa, Ottawa, ON K1H 8M5, Canada

**Keywords:** proximity labeling, AP/MS, BioID, APEX, ChIP, RIP

## Abstract

Proximity-based labeling has emerged as a powerful complementary approach to classic affinity purification of multiprotein complexes in the mapping of protein–protein interactions. Ongoing optimization of enzyme tags and delivery methods has improved both temporal and spatial resolution, and the technique has been successfully employed in numerous small-scale (single complex mapping) and large-scale (network mapping) initiatives. When paired with quantitative proteomic approaches, the ability of these assays to provide snapshots of stable and transient interactions over time greatly facilitates the mapping of dynamic interactomes. Furthermore, recent innovations have extended biotin-based proximity labeling techniques such as BioID and APEX beyond classic protein-centric assays (tag a protein to label neighboring proteins) to include RNA-centric (tag an RNA species to label RNA-binding proteins) and DNA-centric (tag a gene locus to label associated protein complexes) assays.

## Introduction

In the six years following its introduction as a novel method to label neighboring proteins in cells with biotin ^1^, BioID has rapidly established itself as a powerful and complementary approach to classic affinity purification/mass spectrometry (AP/MS)-based interactome mapping. Key advantages include its ability to capture weak/transient interactions that can be lost in standard AP approaches, its applicability to both soluble and insoluble proteins, and the strength of the association of biotin with streptavidin, which allows efficient high-stringency protein extraction and capture methods that help minimize background contaminants. As demonstrated in more than 100 publications to date, BioID can be applied to a wide range of cellular proteins, from transcription factors and signaling molecules to ubiquitin ligases and cytoskeletal components. Although the majority of these studies used cultured cells, the technique has been extended to other model systems, including yeast ^2^, protozoa ^3^, plant protoplasts ^4^, amoebae ^5^, embryonic stem cells ^6^, and xenograft tumors ^7^. The technique has also been combined with lentiviral infection to map synapse-associated protein complexes in intact mouse brain ^8^.

A functionally related method, APEX, was originally developed to facilitate high-resolution imaging of cellular structures by electron microscopy (EM) ^9^ and later extended to proximity labeling of protein complexes in live cells ^10^. A key advantage of APEX over classic BioID is the significantly faster rate of labeling (minutes versus hours). When paired with quantitative proteomic approaches, this higher temporal resolution can facilitate the identification of dynamic changes in protein–protein associations over time or in response to cellular perturbation.

This review will discuss the further evolution of the original BioID and APEX labeling reagents to improve their efficiency and applicability. It will also highlight their integration into organelle-, RNA-, and DNA-centric workflows to aid in the assembly of protein interaction network maps, characterization of ribonucleoproteins (RNPs), and identification of regulatory complexes associated with specific gene loci.

## Biotin-based proximity labeling approaches

**Figure 1.**
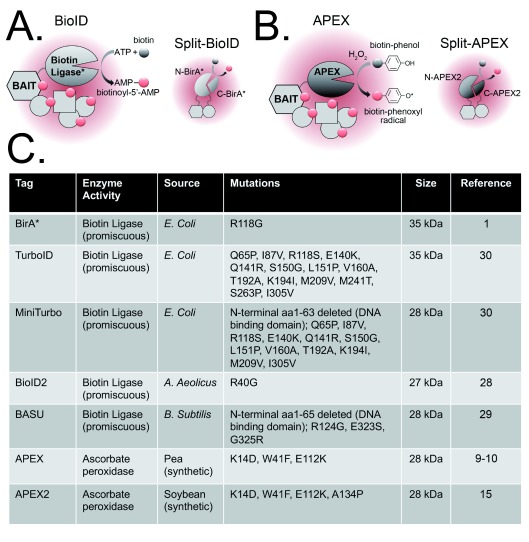
Evolution of biotin-based proximity labeling tags. ( **A**) BioID is based on the expression of a bait protein fused to a mutant biotin ligase that catalyzes the conversion of biotin to biotinoyl-5′-adenosine monophosphate (AMP). This highly reactive form of biotin attaches covalently to accessible lysine residues in neighboring proteins. Split-BioID extends this technique to conditional protein-fragment complementation assays, and N- and C-terminal fragments of the ligase (split at amino acid 256/257 or 140/141) are fused to two different proteins. Activity is regained if (and where) the two proteins associate and promote reconstitution of the ligase. ( **B**) APEX is based on the expression of a bait protein fused to a peroxidase that, in the presence of H _2_O _2_, catalyzes the oxidation of biotin-phenol to a biotin-phenoxyl radical. This activated biotin can attach covalently to electron-rich amino acids (tyrosine and possibly tryptophan, cysteine, and histidine) in neighboring proteins. APEX can also be split into N- and C-terminal fragments (at amino acid 201/202) for protein complementation assays (Split-APEX). ( **C**) Enzymatic tags that have been developed for BioID- and APEX-based proximity labeling approaches.

BioID and APEX are both based on generating a reactive biotin derivative that diffuses from the enzyme’s active site to label proteins in the near vicinity (for a more comprehensive review of these techniques, see 11, 12). The first-generation BioID approach used an engineered *Escherichia coli*-derived biotin ligase (BirA*) with a catalytic site mutation (R118G) that destabilizes retention of the activated biotin molecule (biotinoyl-5′-AMP) ^1^. This dissociates from the ligase and can react with free primary amines of exposed lysine residues in neighboring proteins, resulting in covalent attachment of biotin ( Figure 1A). Cells expressing a bait protein fused to the BirA* tag are incubated with biotin for several hours, after which biotinylated proteins are captured on a streptavidin (or NeutrAvidin) affinity matrix for identification by liquid chromatography-tandem mass spectrometry (LC-MS/MS). As noted above, this technique has been used to map a wide range of interactomes in both small-scale approaches that query a single protein of interest and large-scale network mapping approaches such as analysis of the protein interaction landscape of the centrosome–cilium interface ^13^ and the organization of mRNA-associated granules and bodies ^14^.

APEX is a 27 kDa monomeric ascorbate peroxidase that catalyzes the oxidation of biotin-phenol to the short-lived (<1 ms) biotin-phenoxyl radical in the presence of H _2_O _2_ ( Figure 1B). Reaction with electron-rich amino acids (tyrosine and possibly tryptophan, cysteine, and histidine) in neighboring proteins results in their biotinylation. Following publication of the original reagent, the Ting group employed yeast display evolution to develop the more catalytically active APEX2 ( Figure 1C) ^15^. Cells expressing a bait protein fused to APEX are incubated with biotin-phenol for 30 minutes, followed by a 1-minute exposure to H _2_O _2_ to induce biotinylation. Key advantages of APEX over BioID are its smaller tag size (27 versus 35 kDa; Figure 1C) and speed of labeling (1 minute versus 18–24 hours). Two recent network mapping approaches coupled the time resolution of APEX with quantitative proteomics to spatiotemporally resolve proteins dynamically engaged by G-protein-coupled receptors following ligand-induced activation ^16, 17^. The APEX peroxidase can also catalyze the polymerization and local deposition of diaminobenzidine (DAB), which in turn recruits electron-dense osmium to provide contrast for EM ^18^. This offers the ability to directly couple high-resolution interactome mapping to high-resolution imaging.

Horseradish peroxidase (HRP) can similarly be employed for both EM and proximity labeling but is a larger tag (44 kDa) and not as active in the cytosol and other reducing environments of the cell. It has been used primarily to study cell surface or secretory pathways via antibody- or ligand-based targeting of the enzyme, using techniques such as EMARS (enzyme-mediated activation of radical sources) ^19^ and SPPLAT (selective proteomic proximity labeling using tyramide) ^20^. More recently, HRP was extended to intracellular antibody-based proximity labeling in fixed tissues and cells in a technique called “Biotinylation by Antibody Recognition” ^21^. In this approach, samples are fixed, permeabilized, and stained with the desired primary antibody and species-specific HRP-conjugated secondary antibody, after which they are incubated with biotin-phenol and the biotinylation reaction induced by brief exposure to H _2_O _2_. This approach avoids fusion and overexpression artefacts but requires a monospecific antibody that is not sensitive to fixation artefacts.

As an extension of the applicability of biotin ligase- and peroxidase-based proximity labeling approaches, both BirA* and APEX2 have recently been shown to be amenable to protein-fragment complementation ^22– 24^. This means that they can be divided into N- and C-terminal fragments that will re-form a functional enzyme when brought into close proximity. Thus, *in vivo* protein-fragment complementation assays (PCAs) can be carried out by fusing two proteins of interest to N- or C-terminal BirA* or APEX fragments and expressing them in cells. Association of the target proteins in the presence of biotin (or biotin-phenol and H _2_O _2_) drives enzyme re-formation and subsequent biotinylation of proximal proteins ( Figure 1A and 1B). Although one application of this approach is the validation of binary protein–protein interactions, it can also be used to map conditional interactomes for complexes that form only under specific conditions (for example, phosphorylation of one of the target proteins) and partner-dependent interactomes. The first published split-BioID study mapped interactomes for specific heterodimeric protein phosphatase complexes ^22^, and the second mapped interactomes for the miRISC (microRNA-induced silencing complex) protein Ago2 in complex with two different known binding partners ^23^. These two initial studies show that PCA is supported by splitting the tag at either amino acid (aa) 140/141 ^22^ or 256/257 ^23^, although comparative testing suggests that the aa256/257 split supports a higher reconstituted ligase activity ^23^. For the soybean-derived second-generation enzyme APEX2 ^15^, PCA was supported when the tag was split at aa201/202 ^24^. As with other *in vivo* PCAs, it is important to optimize expression levels of the two fusion proteins to maximize sensitivity and minimize artefacts, and multiple tag conformations may need to be tested to detect enzyme re-formation (that is, different combinations of the fragments fused to either end of the target proteins).

The advantages of biotin-based proximity labeling approaches over classic antibody-based AP/MS are tempered by caveats that need to be taken into account when designing experiments and interpreting results. These include the potential for artefacts owing to the size or placement (or both) of the tag or the level of overexpression (which can affect protein localization/function), and non-specific background biotinylation by free enzyme generated through cleavage or degradation of the fusion protein. Proteins that bind biotin directly, such as the mitochondrial propionyl-CoA carboxylase subunits PCCA and PCCB, also contribute to background noise. Although the strong streptavidin–biotin interaction enables higher-stringency protein extraction and capture workflows that help to minimize non-specific binding of proteins to the affinity matrix, the potential for false positives is not entirely removed.

It should be noted that the high affinity of the streptavidin–biotin interaction, though facilitating the capture of biotinylated proteins, also complicates their elution from the affinity matrix. If proteins that are less biotinylated are eluted more efficiently than highly biotinylated proteins, this will affect both protein identification and quantitation. One option is on-bead digestion, although biotinylated peptides will be left on the affinity matrix. Another is to use an anti-biotin antibody to immunoprecipitate biotinylated proteins or peptides following BioID labeling.

Peroxide-based labeling approaches can also be complicated by the higher hydrophobicity of biotin-phenol compared with biotin, which potentially affects substrate bioavailability in specific cellular locations. Alternate substrates such as biotin-DEAE have been proposed but still need to be tested *in vivo*. There are also concerns that the H _2_O _2_ treatment, though brief, could affect the cellular oxidative status and induce a stress response.

The effective biotinylation radius of the enzyme remains a significant concern for both methods, given that diffusion of the active moieties can lead to biotinylation of proteins whether or not they make direct contact with the bait protein. BioID analysis of different constituents of the structurally well-studied nuclear pore complex estimated a limited labeling radius of about 10 nm for BirA* ^25^. This seems surprisingly small given the half-life of the biotin adenylate ester (on the minute scale) and the relatively long incubation times required for efficient labeling ^11^. Although the labeling radius of APEX has not been measured in a similar fashion, the short half-life of biotin-phenoxyl and EM analysis of diffusion suggest that it is in the range of about 20 nm ^9^. Ongoing attempts to define the labeling radius of both BioID and APEX by network mapping of well-studied protein complexes would help to assess the benefits of one method over the other, depending on, the time and spatial resolution required. Complementary approaches that have been developed to offer higher-specificity labeling include NEDDylation and PUP-IT. NEDDylation is based on the fusion of an engineered version of the NEDD8 E2-conjugating enzyme Ubc12 (NEDDylator) to a bait protein or small molecule, with conjugation of the ubiquitin-like NEDD8 protein tag to prey proteins only occurring by direct attack of their lysine ε-amines upon the thioester in the active site of the Bait-NEDDylator ^26^. PUP-IT is another prokaryote-derived method, developed to identify membrane protein interactions, in which cells co-express a small protein tag (Pup) with a C-terminal Gly-Gly-Glu domain and the Pup ligase (PafA) fused to a bait protein ^27^. In a reaction similar to ubiquitination, PafA catalyzes phosphorylation of the C-terminal Glu in Pup and conjugates it to an exposed lysine-residue side chain on the target protein. Importantly, activated Pup (which cannot diffuse across membranes) is kept bound to the enzyme and thus operates within a more restricted labeling radius. A caveat to PUP-IT is that the enzyme is not as active as APEX and thus a longer labeling time is required.

## Evolution of enzyme tags

The long labeling time required for efficient biotinylation in BioID experiments has limited its use, as it does not permit the capture of an interactome “snapshot” (like AP/MS and APEX) but rather provides datasets that represent the sum of interactions for the target protein over several hours. During this time, the cells may divide and endogenous interactors may be degraded (at different rates), translated, and biotinylated again. To reduce the labeling time required for BioID, significant effort has been directed toward engineering a smaller and more-efficient promiscuous biotin ligase. Having noted that deletion of the N-terminal DNA-binding domain of BirA* adversely affected its ligase activity, the Roux lab used a bioinformatic approach to identify a smaller biotin ligase (27 kDa) from *Aquifex aeolicus* that naturally lacks this domain ^28^. The ligase was humanized, adapted for proximity labeling by mutation of a conserved residue in the catalytic domain (R40G) and dubbed “BioID2” ( Figure 1C) ^28^. In initial testing, BioID2 was found to require significantly less biotin for efficient labeling, and the reduced tag size improved functionality for a bait protein that had exhibited a higher degree of mislocalization with the BirA* tag. A similar approach was taken by the Khavari lab, who developed a new *Bacillus subtilis*-derived promiscuous biotin ligase by identifying and introducing three mutations into its reactive biotin-5′-AMP binding motif (RBAM) ^29^. Removal of the N-terminal DNA-binding domain in this case did not adversely affect activity, and the 28 kDa tag has been dubbed “BASU” ( Figure 1C) ^29^.

The Ting lab recently coupled further mutagenesis of *E. coli*-derived BirA with yeast surface display screening to generate two new tags: TurboID and MiniTurbo ( Figure 1C) ^30^. TurboID is the same size as the original BirA* tag, albeit with 14 mutations in its RBAM that greatly increase its labeling efficiency. MiniTurbo has 12 out of 14 of those RBAM mutations, and the N-terminal DNA-binding domain has been deleted to reduce the tag size to 28 kDa. This does not appear to impact labeling efficiency, as was found for the original BirA*. Both tags were shown to support labeling on a scale of minutes rather than hours. If found to be generally applicable, these new reagents may represent the ideal combination of the catalytic efficiency and temporal resolution of APEX with the simplicity and non-toxicity of BioID. It should be noted that all of the BioID and APEX constructs in Figure 1 have been made freely available to the research community by the authors via Addgene. This, along with the sharing of detailed protocols, is one reason for the rapid and widespread adoption of these powerful techniques and the accumulation of publicly deposited datasets that can be further analyzed for large-scale network analysis and mapping.

Researchers are also developing and sharing more-efficient methods for delivering proximity labeling tags into cells and tissues (lentiviral toolkit assembled by the Gingras lab ^31^) and for carrying out parallel AP/MS and BioID experiments using a single multifunctional tag and the same affinity resin (BirA*-StrepIII MAC-tag designed by the Varjosalo lab ^32^). The deposition of lists of proteins commonly identified in negative control experiments into the searchable online CRAPome contaminant repository ( http://crapome.org) ^33^ has also provided a valuable screening tool to help prioritize hits in BioID datasets for follow-up analysis.

## Biotinylation-based approaches to map ribonucleoprotein complexes

RNPs carry out a large number of essential and functionally diverse roles throughout the cell, and various strategies have been developed over the years to isolate and characterize both their protein and their RNA components. “Protein-centric” approaches capture known RNA-binding proteins (for example, by immunoprecipitation of endogenous protein or AP of an expressed, tagged version) in order to identify associated proteins or RNA or both. In a recent study, BioID was extended to large-scale RNP protein interactome mapping by analyzing the biotinylation profiles of 119 proteins known to be associated with different aspects of mRNA biology ^14^. Network analysis uncovered complexes involved in distinct processes, such as mRNA splicing, and compared the spatial organization of two membrane-less cytoplasmic organelles (stress granules and processing bodies) to highlight distinct and overlapping components.

The identification of RNA species associated with captured protein requires an additional crosslinking step prior to harvesting to preserve the interaction. This technique is known as RNA immunoprecipitation (RIP). The Ting lab recently developed an “APEX-RIP” approach to map transcriptomes for specific subcellular compartments ^34^. Peroxidase was targeted to different regions of the cell—for example, APEX to the nucleus, cytoplasm, mitochondrial matrix, and cytoplasmic face of the endoplasmic reticulum (ER) and HRP to the ER lumen—in the presence of biotin-phenol, followed by chemical crosslinking of protein-RNA, enrichment of biotinylated proteins, and identification of associated RNA using next-generation sequencing (RNA-Seq). A significant advantage of this technique is that it does not require the development of customized purification schemes for each compartment.

RNPs can also be analyzed by using “RNA-centric” approaches, which are based on the capture of a target RNA for the identification of associated proteins by MS. Some use poly(dT) affinity resin to capture all polyadenylated mRNAs for global interactome mapping (for example, RNA interactome capture, or RIC ^35^), whereas others target specific RNAs for pulldown by using short biotinylated complementary oligodeoxyribonucleotides (for example, capture hybridization analysis of RNA targets, or CHART ^36^) or long capture probes tiled across entire target RNA for higher specificity (for example, RNA antisense purification, or RAP ^37^).

**Figure 2.**
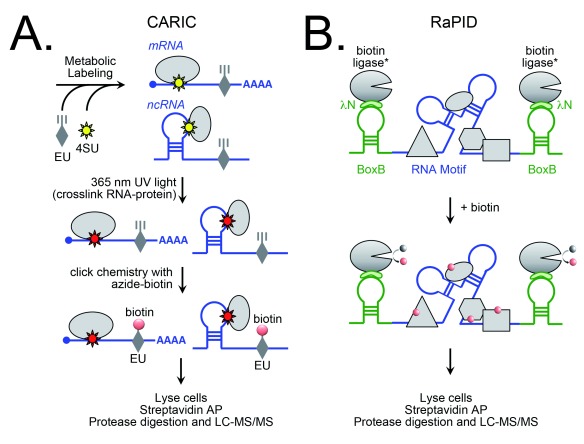
Biotin-based approaches to map ribonucleoprotein complexes. ( **A**) CARIC (click chemistry-assisted RNA interactome capture) is a global screening method for RNA-binding proteins that is based on the incorporation of the uridine analogs 5-ethynyl-uridine (EU) and 4-thiouridine (4SU) into RNA (both messenger RNA and non-coding RNA) during transcription. Exposure to 365 nm ultraviolet (UV) light induces RNA–protein crosslinking via the photoactivatable 4SU. The RNA is then biotinylated by chemosensitive reaction of the alkyne-containing EU with azide biotin via copper(I)-catalyzed azide-alkyne cycloaddition (click chemistry) for efficient capture on a streptavidin affinity matrix. RNA-associated proteins are identified by protease digestion and liquid chromatography-tandem mass spectrometry (LC-MS/MS) analysis. ( **B**) RaPID (RNA–protein interaction detection) is a targeted BioID approach in which an RNA motif of interest is flanked by two bacteriophage lambda BoxB stem loops. Expression of a promiscuous biotin ligase fused to the 22-amino-acid λN peptide, which binds the stem loops with high affinity, results in the recruitment of the fusion protein to the RNA motif and subsequent biotinylation of proteins in close proximity. These proteins then can be captured by streptavidin-based affinity purification (AP) for identification by LC-MS/MS.

A clever variation of RIC—called “RICK”, for “capture of the newly-transcribed RNA Interactome using cliCK chemistry” ^38^—combines metabolic labeling of nascent RNA with the uridine analog 5-ethynyl-uridine (EU), followed by ultraviolet (UV)-induced crosslinking and click chemistry-mediated biotinylation of EU for streptavidin-based capture of labeled RNA. By circumventing the limitations of poly(dT)-based capture (which misses non-polyadenylated mRNAs and non-coding RNAs), this technique enables transcriptome-wide analysis of all RNA species. CARIC (click chemistry-assisted RNA interactome capture) is a similar approach that also incorporates the photosensitive uridine analog 4-thiouridine (4SU), which promotes more-efficient crosslinking using longer-wavelength UV light ( Figure 2A) ^39^.

The BioID-based RaPID (RNA–protein interaction detection) technique is a hybrid RNA-centric method that targets a biotin ligase to a specific RNA sequence to promote biotinylation of associated proteins ^29^. In this approach, the RNA of interest is flanked by two bacteriophage lambda BoxB stem loops, which bind with high affinity to a 22-amino-acid λN peptide fused to the biotin ligase BASU ( Figure 2B). Recruitment of BASU to the RNA motif in the presence of biotin promotes biotinylation of proximal proteins, which can be captured with streptavidin for MS-based identification. The technique was used to assess the effect of disease-related point mutations on protein binding and to identify proteins that associate with Zika virus RNA ^29^. An article detailing a similar approach (RNA-BioID), based on the incorporation of MS2 stem loops into nascent RNA and the expression of BirA* fused to the MS2 coat protein (MCP) for proximal protein labeling, was recently deposited on bioRxiv ^40^. Given the success of BioID in mapping multiprotein complexes, it is likely that these types of RNA-targeted proximity labeling approaches will become popular methods for characterizing RNPs.

## Targeted analysis of chromatin-associated protein complexes

Another area of research that would benefit greatly from increased sensitivity and specificity is the mapping of transcriptional regulatory complexes on chromatin. Gene expression is controlled by the assembly of unique combinations of transcriptional regulatory proteins on specific regions of DNA, which can result in either activation or repression of the target gene. Chromatin immunoprecipitation (ChIP) is a highly popular technique that has been used to identify the region(s) of the genome with which a particular transcription factor associates. Chemical crosslinking (usually formaldehyde based) is followed by shearing of genomic DNA into smaller fragments and immunoprecipitation of the protein of interest (endogenous or tagged). The crosslinks are reversed to release the associated DNA fragments, which can then be identified by next-generation sequencing (ChIP-Seq). An alternate method for mapping protein–DNA associations is DamID, which is based on expression of the protein of interest fused to a DNA adenine methyltransferase and subsequent identification of adenine-methylated DNA fragments ^41^. A limitation of both strategies is that they provide information about DNA interactions only for the protein chosen for capture (or methyltransferase-based labeling), although bioinformatic analysis of the overlap of datasets collected for multiple DNA-binding proteins can help to identify patterns and potential complexes.

**Figure 3.**
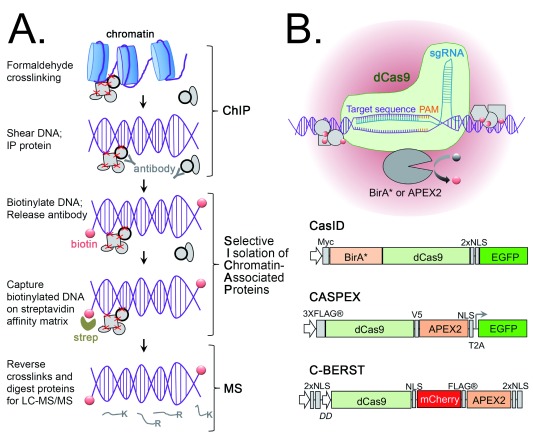
Biotin-based approaches to map chromatin-associated protein complexes. ( **A**) ChIP-SICAP (chromatin immunoprecipitation combined with selective isolation of chromatin-associated proteins) is a modified ChIP method that was developed to increase the specific identification of chromatin-associated complexes for the target protein of interest. It starts with formaldehyde crosslinking, DNA shearing, and immunoprecipitation (IP) of a specific bait protein. The DNA fragments are then end-labeled with biotin—via treatment with terminal deoxynucleotide transferase (TdT) and biotin-ddUTP—for capture on a streptavidin affinity matrix and stringent wash steps prior to liquid chromatography-tandem mass spectrometry (LC-MS/MS)-based protein identification. ( **B**) CRISPR/Cas9 (clustered regularly interspaced short palindromic repeats/Cas9)-based gene targeting has been harnessed for BioID and APEX proximity labeling approaches via co-expression of a specific single-guide RNA (sgRNA) with a catalytically dead Cas9 enzyme fused to either BirA* (CasID) or APEX2 (CASPEX and C-BERST). Recruitment of the fusion protein to a specific gene locus promotes the biotinylation of proteins in close proximity, which then can be captured on a streptavidin affinity matrix for identification by LC-MS/MS.

ChIP-MS strategies have been developed to enable the parallel identification of both DNA regions and proteins that co-precipitate with a target protein. Captured proteins can be digested for analysis after elution (ChIP-MS ^42, 43^) or directly on the affinity matrix beads (rapid immunoprecipitation mass spectrometry of endogenous proteins, or RIME ^44^). Caveats can include high levels of background contaminants, masking of low-abundance hits by the large number of antibody peptides, and identification of non-chromatin-associated complexes and DNA “hitchhikers” (proteins not directly associated with the target protein but bound further along the DNA strand). ChIP-SICAP (ChIP combined with selective isolation of chromatin-associated proteins) was recently developed to help distinguish chromatin-associated from non-chromatin-associated interactors for a target protein ^45^. It starts out as a standard ChIP experiment (capture of target protein and associated crosslinked complexes) but then adds a second purification step to specifically enrich chromatin-associated complexes for MS analysis. This is based on terminal deoxynucleotide transferase (TdT)-mediated end labeling of DNA with biotinylated nucleotides and capture on streptavidin beads ( Figure 3A).

In order to identify, in a non-biased fashion, transcriptional regulatory complexes that associate with a specific gene locus *in vivo* (a DNA-centric approach), target DNA can be directly captured for the identification of associated proteins using MS. This is called “Reverse ChIP”, and strategies such as PICh (proteomics of isolated chromatin segments) ^46^ and HYCCAPP (hybridization capture of chromatin-associated proteins for proteomics) ^47^ are based on sequence-specific hybridization and capture of nucleic acid probes. The advent of the CRISPR/Cas9 (clustered regularly interspaced short palindromic repeats/Cas9) system provided another option for targeted studies of the landscape surrounding specific regions of DNA.

Originally developed to facilitate *in vivo* gene editing, CRISPR/Cas9 is based on the targeting of the Cas9 endonuclease to a specific genomic sequence by co-expression of a single-guide RNA (sgRNA). Mutagenesis of the Cas9 enzyme to render it catalytically dead (dCas9) allows it be targeted to a specific gene locus without inducing double-strand breaks. Fusion of regulatory proteins or fluorophores to dCas9 takes advantage of this and has enabled techniques such as sequence-specific gene regulation, epigenetic editing and visualization of gene loci in live cells. (for review, see 48). The first methods that took advantage of dCas9 for AP/MS-based proteome mapping include CRISPR-ChAP-MS (CRISPR-based chromatin affinity purification with mass spectrometry) ^49^, enChIP (engineered DNA-binding molecule-mediated chromatin immunoprecipitation) ^50^, and CLASP (Cas9 locus-associated proteome) ^51^. All are based on targeting of dCas9 to a specific gene locus via co-expression with an sgRNA, followed by chemical crosslinking, DNA shearing, and AP/MS of the tagged dCas9. The related CAPTURE (CRISPR affinity purification *in situ* of regulatory elements) method introduces a biotin acceptor peptide into dCas9 and co-expresses it with both a target-specific sgRNA and wild-type BirA ^52^. The ligase specifically biotinylates the peptide fused to dCas9 for high-affinity streptavidin-mediated capture and MS analysis.

Although the crosslinking step in these AP/MS-based approaches helps to stabilize chromatin-associated protein complexes for purification, the increased sensitivity and more efficient extraction and AP strategies offered by proximity labeling approaches inspired the combination of BioID and APEX with CRISPR/dCas9-based strategies ( Figure 3B). CasID employs a classic BioID approach by fusing dCas9 to BirA* ^53^. Co-expression of this fusion protein with a specific sgRNA in the presence of biotin leads to the biotinylation of proteins in close proximity to the targeted gene locus. The system was tested by probing repetitive telomeric, major satellite, and minor satellite DNA regions.

In a similar approach, dCas9 has been fused to APEX2. C-BERST (dCas9-APEX2 biotinylation at genomic elements by restricted spatial tagging) was tested by targeting dCas9-APEX2 to telomeric and centromeric regions, finding good agreement with results from previous BioID and PICh experiments (plus unique hits) ^54^. The GLoPro (genomic locus proteomics) technique, which uses a dCas9-APEX2 fusion protein called CASPEX ( Figure 3B), was applied to the analysis of proteins associated with either hTERT or MYC promoter regions ^55^. This demonstrates the applicability of dCas9-mediated proximity labeling to single loci (not limited to the analysis of repetitive regions). The authors note the caveat that identification only confirms association with a locus, not locus specificity (that is, the same complex may bind to other genomic regions); however, future bioinformatic analysis of large collections of single loci datasets will help to identify patterns and define specificities.

## Future directions

In the future, it will be interesting to extend the dCas9-based proximity labeling strategies to the dynamic analysis of gene loci during development or in response to perturbations that affect their transcriptional regulation. Given that the functional CRISPR/Cas9 gene editing system is now routinely used to fuse affinity tags and fluorophores directly to endogenous proteins, it will likely be employed in a similar fashion to introduce biotin ligase or APEX (provided that the constitutive presence/position of the enzyme tag does not affect the protein’s function). This has already been demonstrated in protozoa ^56, 57^ and should be equally applicable in mammalian cells. This would also facilitate the tagging of target proteins for direct analysis in mouse models. An alternative approach (particularly if there are concerns that the tag is interfering with protein function) would be to genetically fuse a biotin acceptor tag to an endogenous protein and virally infect a wild-type BirA expression plasmid to promote its specific biotinylation.

Another area of research that would pair well with proximity labeling is *in vivo* expression of nanobodies (single-domain camelid monoclonal antibodies). Because they can be genetically encoded, nanobodies have already been expressed in live cells fused to fluorophores to enable live imaging of endogenous proteins. Their small size compared with that of conventional antibodies (about 15 kDa versus about 150 kDa) places the fluorophore closer to the target protein, which offers a much higher spatial resolution ^58^. A recent study fused secondary nanobodies (anti-mouse IgG and anti-rabbit IgG) to APEX2 to assess their applicability for *in vitro* enzyme-linked immunosorbent assays (via oxidation of the Amplex Red Ultra substrate) ^59^. The fusions expressed well in bacteria and the recombinant enzyme was active, suggesting that this approach may be applicable to cell-based systems. Although the generation and validation of primary nanobodies can be expensive and laborious, recent advances in yeast surface display of synthetic libraries ^60^ and an expanding range of commercial services offer alternatives to buying a llama.
